# Osimertinib and pterostilbene in EGFR-mutation-positive non-small cell lung cancer (NSCLC)

**DOI:** 10.7150/ijbs.32889

**Published:** 2019-09-07

**Authors:** Jillian Wilhelmina Paulina Bracht, Niki Karachaliou, Jordi Berenguer, Carlos Pedraz-Valdunciel, Martyna Filipska, Carles Codony-Servat, Jordi Codony-Servat, Rafael Rosell

**Affiliations:** 1 Pangaea Oncology, Laboratory of Molecular Biology, Quirón-Dexeus University Institute, Barcelona, Spain; 2 Instituto Oncológico Dr Rosell (IOR), University Hospital Sagrat Cor, QuironSalud Group, Barcelona, Spain; 3 Institut d'Investigació en Ciències Germans Trias i Pujol, Badalona, Spain; 4 Institut Català d'Oncologia, Hospital Germans Trias i Pujol, Badalona, Spain

**Keywords:** Pterostilbene, NSCLC, osimertinib, therapy resistance

## Abstract

Monotherapy with epidermal growth factor receptor (EGFR) tyrosine kinase inhibitors (TKIs) still leads to incomplete responses in most EGFR-mutation positive non-small cell lung cancer (NSCLC) patients, often due to acquired resistance through activation of parallel compensatory pathways. We have previously shown that co-targeting EGFR, signal transducer and activator of transcription 3 (STAT3), and Src-yes-associated protein 1 (YAP1) was highly synergistic in vitro and in vivo. In the present study, we treated EGFR-mutation positive cell lines with the combination of osimertinib plus a natural compound, pterostilbene, which has been reported to abrogate Src and STAT3 activation.

**Methods**: Cell viability assays and immunoblotting were performed to reveal the mechanisms of action of pterostilbene, osimertinib and pterostilbene plus osimertinib in five EGFR-mutation positive NSCLC and one triple negative breast cancer (TNBC) cell lines.

**Results**: Osimertinib plus pterostilbene yielded synergistic effects in all EGFR-mutation positive NSCLC cell lines investigated. Surprisingly, pterostilbene alone did not inhibit, nor downregulate Src phosphorylation in the EGFR-mutation positive NSCLC cell lines or the TNBC cell line, MDA-MB-231. However, the double combination of osimertinib plus pterostilbene reversed the osimertinib-induced STAT3, YAP1, and CUB domain-containing protein-1 (CDCP1) phosphorylation and slightly suppressed Src phosphorylation in PC9 and H1975 cells.

**Conclusion**: The results of this study indicate that pterostilbene may be used to abrogate the activated resistance pathways of single osimertinib treatment in EGFR-mutation positive NSCLC. Future studies should focus on in vivo translation and confirmation of these results.

## Introduction

Epidermal growth factor receptor (EGFR) mutations in non-small cell lung cancer (NSCLC) patients were discovered in 2004 1. To date, monotherapy with EGFR tyrosine kinase inhibitors (TKIs) still leads to incomplete responses in 95% of patients 2, often due to intrinsic or acquired resistance. Relevant signaling network- and crosstalk changes after EGFR blockade are underappreciated, including hyperactivation of signal transducer and activator of transcription 3** (**STAT3) 3-7. Ongoing and previously published research indicates that gefitinib, afatinib and osimertinib TKI treatments are unable to inhibit STAT3 activation, and lead to parallel compensatory activation of the Src-yes-associated protein 1 (YAP1) signaling pathway 8-10. We have previously shown that co-targeting EGFR, STAT3 and Src-YAP1 was highly synergistic in vitro and in vivo. We also found that several receptor tyrosine kinases (RTKs) and non-RTKs are upregulated at baseline or after treatment with gefitinib or osimertinib, limiting their therapeutic efficacy 8-11.

The genetic or pharmacologic inhibition of Src family kinases (SFKs) or YAP1 diminishes the phosphorylation of the RTK AXL and the transmembrane protein CUB domain-containing protein-1 (CDCP1) 10. When overexpressed, both of these proteins are related to worse survival outcomes in patients treated with single EGFR TKIs. The combination of EGFR TKIs with a multikinase inhibitor, that inhibits janus kinase 2 (JAK2), Src and focal adhesion kinase (FAK), abrogates not only STAT3, but also YAP1 and SFKs activation and downregulates AXL and CDCP1 expression 10.

**Figure 1:**
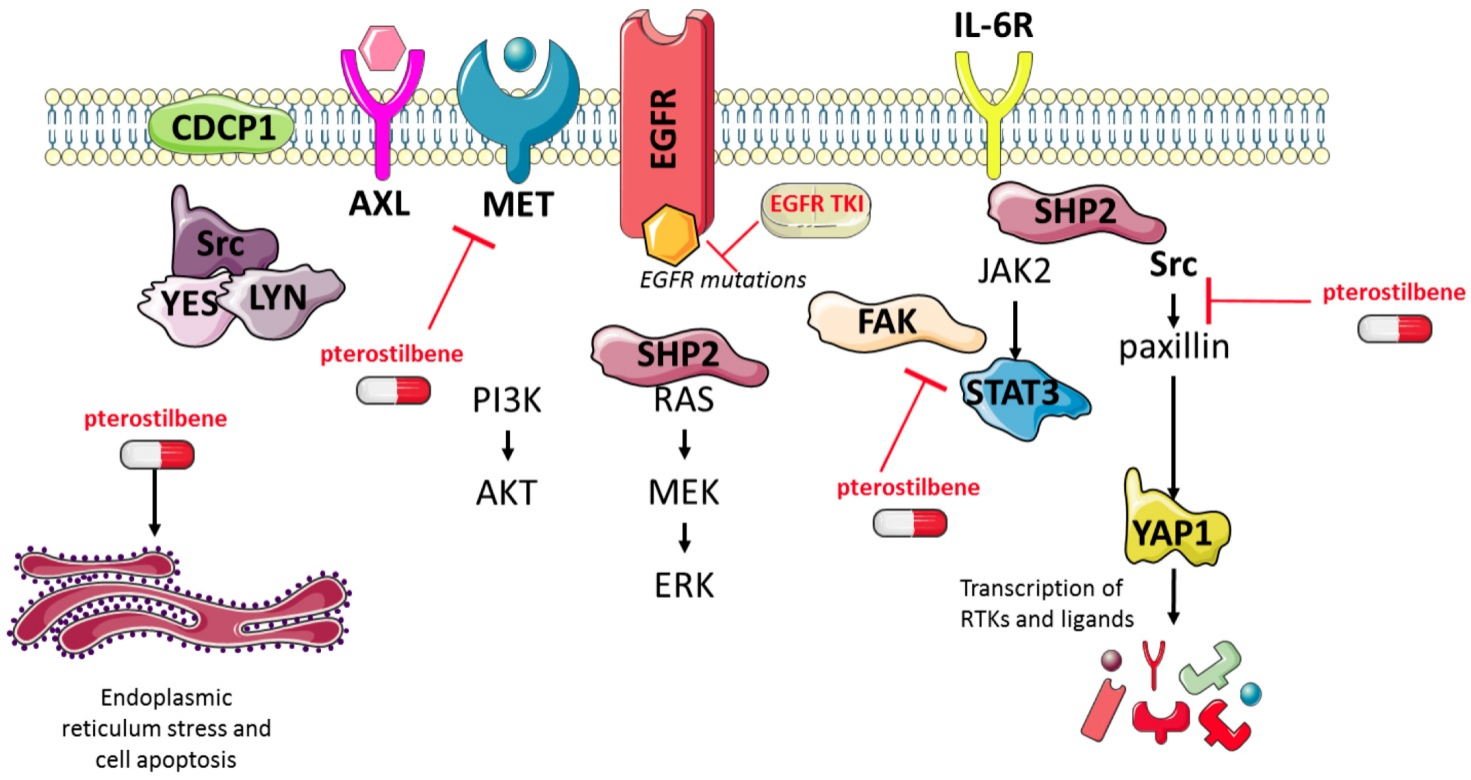
** The effects of pterostilbene^1^ and EGFR TKIs on RTKs and downstream components.** EGFR TKIs block signaling of the EGFR receptor and its downstream pathways. Previous research has shown that this process causes hyper-activation of compensatory pathways, such as STAT3 and Src-YAP1. Consequently, this leads to an up-regulation of RTKs and non-RTKs (e.g. MET, CDCP1), and therapy resistance. Pterostilbene has been shown to inhibit pathways involved in TKI resistance, such as STAT3 and Src-YAP1. Moreover, it was able to inhibit MET, and cause ER-stress and apoptosis. CDCP1: CUB domain-containing protein-1; EGFR: epidermal growth factor receptor; ER: endoplasmic reticulum; FAK: focal adhesion kinase; JAK2: janus kinase 2; RTKs: receptor tyrosine kinases; STAT3: signal transducer and activator of transcription 3; TKIs: tyrosine kinase inhibitors; YAP1: Src-yes-associated protein 1.

Pterostilbene (3,5-dimethoxy-4'-hydroxy-trans-stilbene) is a stilbene of the family of phytoalexin compounds, found in in blueberries and Pterocarpus marsupium (PM) heartwood. It is structurally similar to resveratrol, a compound found in red wine that has comparable antioxidant, anti-inflammatory, and anti-carcinogenic properties. Due to the presence of two methoxyl groups, pterostilbene has increased lipophilic and oral absorption and therefore increased bioavailability compared to resveratrol 12. It has been shown that pterostilbene has apoptotic and anti-proliferative effects in solid tumors 13, including EGFR-mutation positive NSCLC 14. In triple negative breast cancer (TNBC), pterostilbene abolished the activation of Src, FAK, Paxillin and STAT3. Moreover, by altering mainly the Src-mediated signaling pathway pterostilbene suppressed the metastatic potential of TNBC cells. It was also found to decrease the levels of mesenchymal markers, amongst which MET 15. Pterostilbene also causes endoplasmic reticulum (ER) stress and consequently leads to apoptosis 14. In addition, pterostilbene was shown to be safe in patients, even at high doses 16-18. Henceforth, we posit that the combination of pterostilbene plus an EGFR TKI could substantially improve the outcome of single EGFR TKIs in EGFR-mutation positive NSCLC (**Figure 1**). In this study we explored whether pterostilbene inhibits compensatory osimertinib-induced signaling pathways, and if the combination can optimize the upfront therapy of EGFR-mutation positive NSCLC cells.

## Methods

### Chemicals and reagents

Human lung adenocarcinoma PC9 cells, harboring EGFR exon 19 deletion and 11-18 cells, harboring EGFR exon 21 L858R mutation were provided by F. Hoffmann-La Roche Ltd. (Basel, Switzerland), and by Dr. Mayumi Ono, (Kyushu University, Fukuoka, Japan), respectively. EGFR exon 19 deletion positive HCC4006 and HCC827 cells were purchased from the American Type Culture Collection (ATCC). The H1975 cell line, harboring both EGFR exon 21 L858R and resistant T790M mutation as well as the TNBC MBA-MB-231 cell line were purchased from ATCC. All cell lines were maintained in RPMI (Roswell Park Memorial Institute medium) 1640 supplemented with 1% penicillin/streptomycin/glutamine (Gibco) and 10% fetal bovine serum (FBS; Gibco) in a 5% CO_2_ 37°C cell culture incubator and routinely evaluated for mycoplasma contamination.

The primarily used pterostilbene compound (hereafter referred to as pterostilbene^1^) was purchased from Amazon.com, Inc. (Catalog: B00BFUJ04Q), the second pterostilbene compound (hereafter referred to as pterostilbene^2^) was bought from Sigma Aldrich/Merck KGaA, (Darmstadt, Germany, Catalog: P1499-10MG) and osimertinib was bought from Selleck Chemicals (Houston, TX, U.S.). Drugs were prepared in dimethylsulfoxide (DMSO) at a concentration of 10-100 mM stock solutions and stored at -20°C. Further dilutions were made in culture medium to final concentration before use. All antibodies used in our study, including dilution and company catalog number can be found in **Table 1**.

### Cell viability assay

Cells were seeded in 96-well plates at the following densities: 1 x 10^3^ (PC9 and H1975) 1.5 x 10^3^ (HCC4006, HCC827 and 11-18), and incubated for 24h, as previously described 8. Cell viability was assessed using the 3-[4,5-dimethylthiazol-2-yl]-2,5-diphenyltetrazolium bromide (MTT) assay (Sigma Aldrich, St Louis, MO, U.S). Cells were treated with serial dilutions of the drugs. For the half maximal inhibitory concentration (IC_50_) determination, MTT viability assays were performed using pterostilbene doses ranging from 0-150 μM. To determine the combined effect of pterostilbene and osimertinib, drug doses in viability assays were as follows: H1975 and HCC4006 cells were treated with osimertinib ranging from 0-90 nM, and pterostilbene ranging from 0-180 μM, or with the combination of both. PC9 and HCC827 cells were treated with osimertinib ranging from 0-120 nM, and pterostilbene ranging from 0-120 μM, or with the combination of both. 11-18 was treated with osimertinib ranging from 0-330 nM, and pterostilbene ranging from 0-120 μM, or with the combination of both. After 72 h of treatment incubation, 0.5 mg/ml of MTT was added to the medium in the wells for 2 h at 37 °C and formazan crystals in viable cells were solubilized with 100 μl DMSO and spectrophotometrically quantified using a microplate reader (Varioskan Flash; Thermo Fisher Scientific, Waltham, MA, U.S) at 565 nm of absorbance. Fractional survival was then calculated as percentage to control cells. Data of combined drug effects were subsequently analyzed by the Chou and Talalay method 19, 20. Combination Index (CI) values <1, =1 and >1 indicated synergism, additive effect and antagonism, respectively. All experiments were performed in biological triplicates.

### Western blotting

For immunoblotting experiments, 1.5 million cells were seeded in T75 flasks (Starstedt, Newton, U.S). The next day, PC9, H1975 and MDA-MB-231 cells were either treated with increasing doses of pterostilbene (10, 20 and 50 μM), or with 20 nM osimertinib, 10 μM pterostilbene and a combination of both. Untreated cells received an equivalent dose of vehicle (DMSO). After 24 h cells were washed with cold PBS and re-suspended in ice-cold radioimmunoprecipitation assay (RIPA) buffer (50 mM Tris- hydrochloric acid in pH 7.4, 1% Nonidet P-40, 0.5% sodium deoxycholate, 0.1% sodium dodecyl sulfate [SDS], 150 mM sodium chloride, 1 mM ethylenediaminetetraacetic acid, 1 mM sodium vanadate and 50 mM sodium fluoride) containing protease inhibitor mixture (Roche). Following cell lysis by sonication and centrifugation at 18620x g for 10 min at 4 °C, the resulting supernatant was collected as the total cell lysate. Briefly, the lysates containing 45 μg proteins were electrophoresed on 10% SDS-polyacrylamide gels (Life Technologies, Carlsbad, CA, U.S) and transferred to polyvinylidene difluoride (PVDF) membranes (Bio-Rad laboratories Inc., Hercules, CA, U.S). Membranes were blocked in Odyssey blocking buffer (Li-Cor Biosciences, Lincoln, NE, U.S). All target proteins were immunoblotted with appropriate primary and horseradish peroxidase (HRP)-conjugated secondary antibodies. Chemiluminescent (HRP-conjugated) bands were detected in a ChemiDoc MP Imaging System (Bio-Rad laboratories Inc.). β-actin was used as an internal control to confirm equal gel loading. Experiments were performed in biological triplicates with similar results, and representative blots were shown.

## Results

### Sensitivity of EGFR-mutation positive cells to pterostilbene and the combination of osimertinib plus pterostilbene

**Figure 2:**
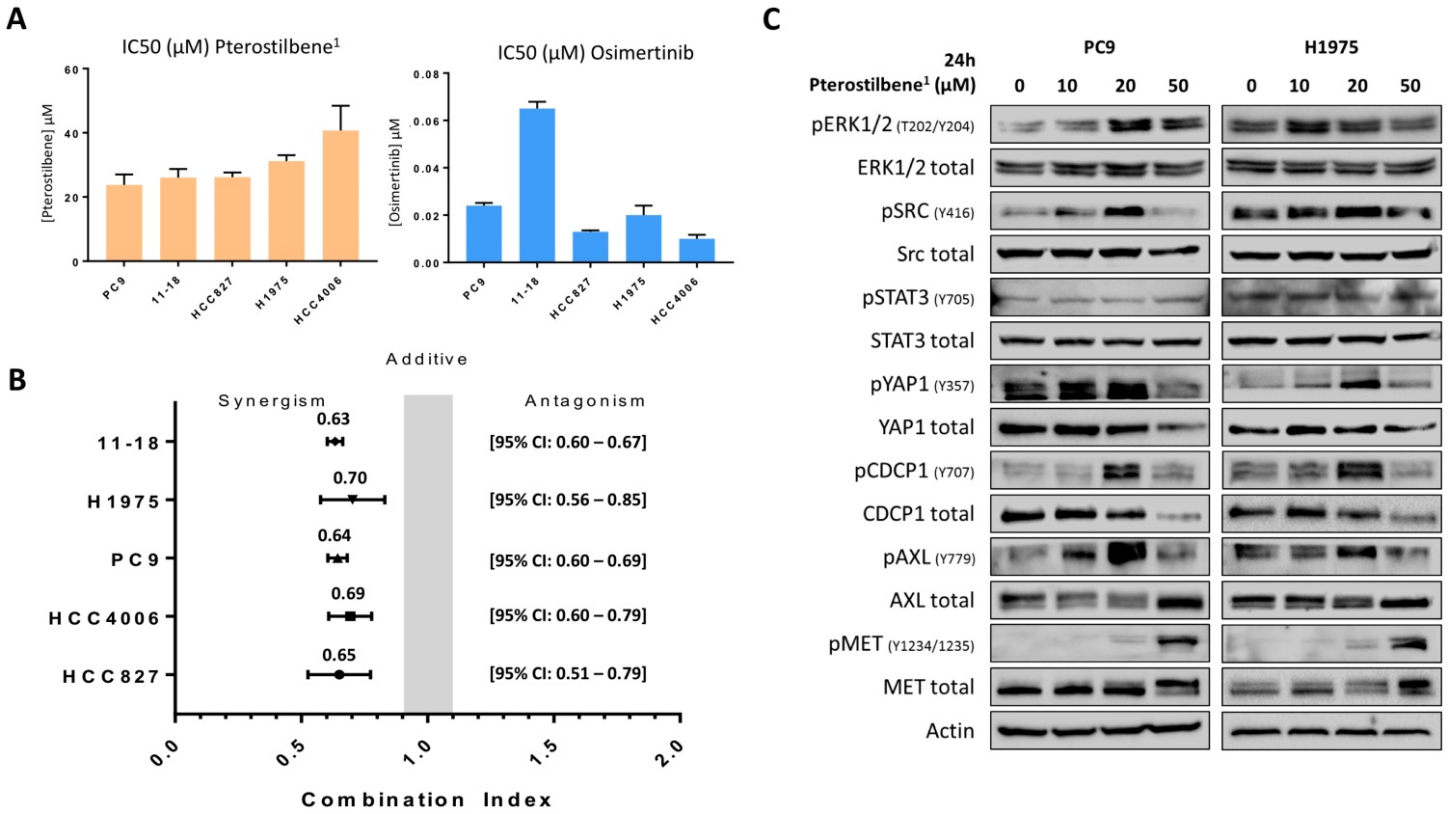
** The effects of single and combined osimertinib and pterostilbene^1^ treatment. A**: MTT cell viability assays were performed in 5 different EGFR-mutation positive NSCLC cell lines (PC9, H1975, 11-18, HCC4006 and HCC827), to determine the effects of pterostilbene^1^ on cell viability, and to determine the IC_50_ in each cell line. Results of previously performed experiments with osimertinib in the same cell lines are also shown. Experiments were performed in biological triplicates, and the average and standard deviations are shown. **B**: In all cell lines MTT cell viability assays were performed to explore the effect of combined osimertinib and pterostilbene^1^ treatment. Combination indexes were calculated based on the Chou and Talalay method, and values <1, =1 and >1 indicate synergism, additive effect and antagonism, respectively. Experiments were performed in biological triplicates, and the averages and 95% CIs are shown. **C:** PC9 and H1975 cells were treated with different concentrations of pterostilbene^1^ for 24 h. Untreated cells received an equivalent dose of vehicle (DMSO). Cell lysates were used for immunoblotting, and the effect of pterostilbene^1^ treatment on downstream components was explored. Experiments were performed in biological triplicates with similar results, and a representative blot is shown. DMSO: dimethylsulfoxide; EGFR: epidermal growth factor receptor; IC_50_: the half maximal inhibitory concentration; MTT: 3-[4,5-dimethylthiazol-2-yl]-2,5-diphenyltetrazolium bromide; NSCLC: non-small cell lung cancer; 95%CI: 95% confidence interval.

To confirm the effects of pterostilbene on cell growth, cell viability assays were performed in 5 different EGFR-mutation positive NSCLC cell lines: PC9, H1975, HCC827, HCC4006 and 11-18. After establishing the half maximal inhibitory concentration (IC_50_) of pterostilbene^1^ in all cell lines (**Figure 2A**), it could be concluded that single pterostilbene^1^ treatment was not able to induce anti-proliferative effects in these cell lines, with IC_50_s ranging from 23.8 to 40.7 μM. However, when combining pterostilbene^1^ with the third-generation TKI osimertinib (of which IC_50_s were previously determined, and can be found in **Figure 2A**), we obtained synergistic results in all EGFR-mutation positive NSCLC cell lines investigated, with a combination index (CI) ranging from 0.63 to 0.70 (**Figure 2B**). These results indicate a synergistic interaction between pterostilbene^1^ and osimertinib in these cell lines.

### Correlation between signaling nodes and cellular responses to pterostilbene or to the combination of osimertinib plus pterostilbene

To determine which signaling nodes are being abrogated by pterostilbene^1^ treatment, PC9 and H1975 cells were treated with different concentrations of pterostilbene^1^ for 24h. Cell lysates were consequently used for immunoblotting and revealed different mechanisms of action of pterostilbene^1^ on (non-)RTKs, as can be seen in **Figure 2C**. An increase of ERK, CDCP1, STAT3 and Src phosphorylation was observed upon different concentrations of pterostilbene^1^ treatment. In contrast, both total and phosphorylated YAP1 were inhibited by pterostilbene^1^ at higher concentrations. It can also be seen that phosphorylation of Src, YAP1, pCDCP1 and pAXL are correlated, indicating an interrelationship between thse signaling nodes as previously described 10. The increase in Src and MET phosphorylation compared to baseline after 24h treatment with 10 or 20 μM pterostilbene^1^ is opposite from previously published results, in which pterostilbene was reported to be a Src and MET inhibitor in TNBC 15.

**Figure 3:**
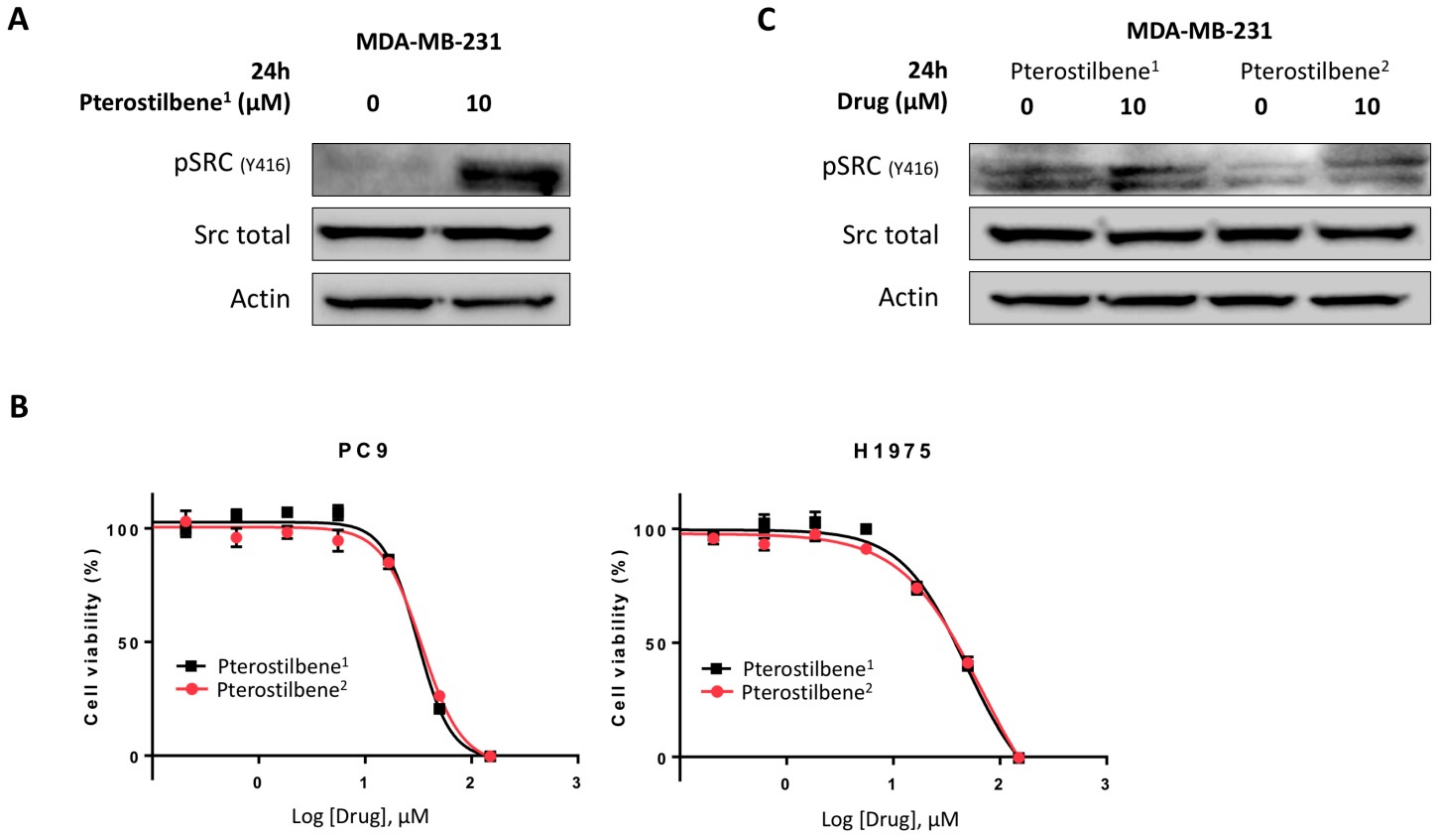
** Unraveling the mechanisms of action of pterostilbene in TNBC and NSCLC cell lines. A:** The TNBC cell line MDA-MB-231 was treated with 10 μM pterostilbene^1^ for 24 h. Untreated cells received an equivalent dose of vehicle (DMSO). Cell lysates were used for immunoblotting, and the effect of pterostilbene^1^ treatment on (phosphorylation of) Src was explored. Experiments were performed in biological triplicates with similar results, and a representative blot is shown. **B:** MTT cell viability assays were performed in 2 different EGFR-mutation positive NSCLC cell lines (PC9 and H1975), to compare the effects of the two different pterostilbene compounds (pterostilbene^1^: Amazon; pterosilbene^2^: Sigma Aldrich) on cell viability. Experiments were performed in biological triplicates with similar results, and a representative plot is shown. **C:** To confirm a similar mechanism of action of the two pterostilbene compounds, MDA-MB-231 cells were treated with 10 μM pterostilbene^1^ or 10 μM pterostilbene^2^ for 24 h. Untreated cells received an equivalent dose of vehicle (DMSO). Cell lysates were used for immunoblotting, and the effect of pterostilbene treatment on (phosphorylation of) Src was explored. Experiments were performed in biological triplicates with similar results, and a representative blot is shown. DMSO: dimethylsulfoxide; EGFR: epidermal growth factor receptor; MTT: 3-[4,5-dimethylthiazol-2-yl]-2,5-diphenyltetrazolium bromide; NSCLC: non-small cell lung cancer; TNBC: triple negative breast cancer.

To explore these contradictory results, the activity of pterostilbene^1^ in the MBA-MB-231 TNBC cell line was determined. Cells were treated with 0 or 10 μM pterostilbene^1^ for 24 h, and Src activation was explored. Surprisingly, instead of inhibiting Src phosphorylation, pterostilbene^1^ caused an induction of Src phosphorylation (**Figure 3A**). To confirm this, we purchased pterostilbene from a different supplier (pterostilbene^2^), and cell viability experiments were performed to compare the two drugs. In **Figure 3B** it can be seen that the two pterostilbene compounds have nearly identical anti-proliferative effects in two EGFR-mutation positive NSCLC cell lines. To verify these results in the TNBC cell line, MBA-MB-231 cells were treated with both pterostilbene compounds. The cell lysates were consequently used for immunoblotting, and again revealed similar mechanisms of action, as can be seen in **Figure 3C**. Both compounds clearly induce Src phosphorylation.

**Figure 4:**
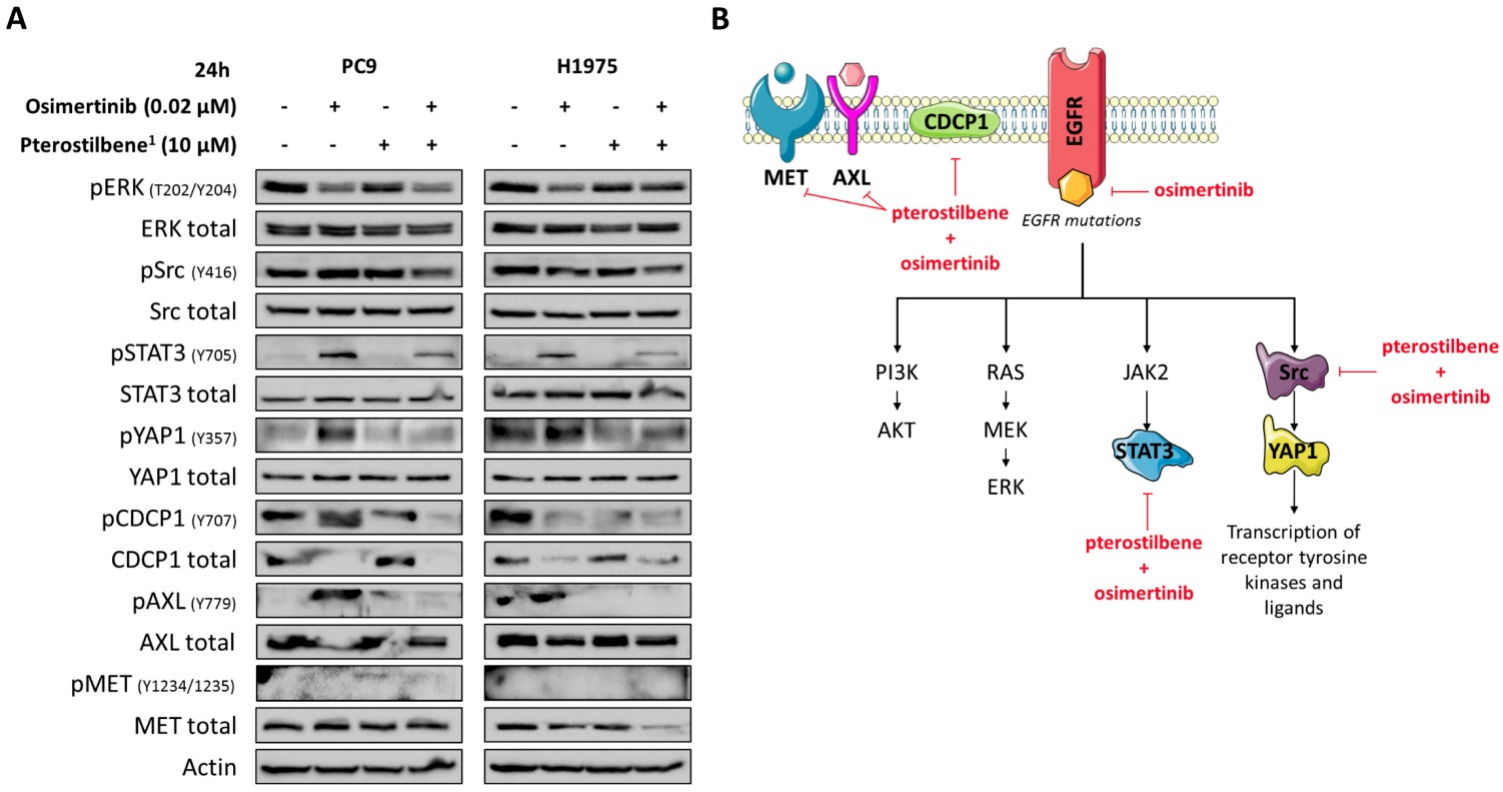
** The effects of combined osimertinib and pterostilbene^1^ treatment in NSCLC cell lines. A:** Two EGFR-mutation positive NSCLC cell lines (PC9 and H1975) were treated with single osimertinib (0.02 μM), single pterostilbene^1^ (10 μM) or with the combination. Untreated cells received an equivalent dose of vehicle (DMSO). Cell lysates were used for immunoblotting and changes in RTKs and non-RTKs upon the different treatments were investigated in the two cell lines. Experiments were performed in biological triplicates with similar results, and a representative blot is shown. **B:** Our model: osimertinib blocks the signaling of the EGFR receptor and its downstream pathways. Our previous research has shown that this process causes hyperactivation of compensatory signaling nodes, including STAT3 and Src-YAP1. This leads to an up-regulation of RTKs and non-RTKs (e.g. MET, CDCP1), and ultimately to therapy resistance. The combination of pterostilbene with osimertinib abrogates the osimertinib-induced activation of STAT3, YAP1, CDCP1 and moderately abrogates Src activation. MET expression is also inhibited with the double combination in the H1975 cell line. CDCP1: CUB domain-containing protein-1; DMSO: dimethylsulfoxide; EGFR: epidermal growth factor receptor; NSCLC: non-small cell lung cancer; RTKs: receptor tyrosine kinases; STAT3: signal transducer and activator of transcription 3; YAP1: Src-yes-associated protein 1.

Although in our study pterostilbene was not able to inhibit Src phosphorylation, its effects on YAP1, Src, CDCP1 and AXL phosphorylation in EGFR-mutation positive cells led us to explore the combination of osimertinib plus pterostilbene^1^. As we have previously reported, osimertinib alone attenuated ERK activation in both cell lines, but induced CDCP1 and Src activation in PC9 cells, and STAT3, YAP1 and AXL activation in both PC9 and H1975 cells (**Figure 4A**). In contrast, STAT3, YAP1 and AXL phosphorylation are completely abrogated when treated with single pterostilbene^1^. MET phosphorylation is slightly activated upon single pterostilbene^1^ treatment in the PC9 cell line. While in both cell lines CDCP1 activation is inhibited compared to baseline levels, Src phosphorylation is not abrogated with single pterostilbene^1^ treatment. Osimertinib plus pterostilbene^1^ reversed the osimertinib-induced STAT3 and YAP1 phosphorylation, abolished CDCP1 and AXL activation and decreased Src phosphorylation. In the PC9 cell line, a restoration of MET phosphorylation levels to baseline was also observed with the double combination. The above results indicate that osimertinib treatment activates parallel compensatory pathways, which can be abrogated by combined osimertinib plus pterostilbene treatment.

## Discussion

EGFR-mutation-positive NSCLC cells co-express RTKs and non-RTKs, especially, Src, YES and FAK, which cannot be inhibited by single EGFR inhibitors, including the third-generation EGFR TKI osimertinib 8-10, 21-23. EGFR, SFK and FAK concomitant inhibition enhances osimertinib activity and suppresses resistance 21-23. We have confirmed our previous findings that the third generation TKI osimertinib instead of inhibiting, induces STAT3 and YAP1 (PC9 and H1975) in addition to Src and CDCP1 phosphorylation (only PC9) in the PC9 and H1975 EGFR-mutation positive NSCLC cell lines 8-10.

Pterostilbene is a natural nutrient from blueberries that can be safely used in high doses 16-18. Recent studies suggest that pterostilbene modulates the hallmarks of aging, like oxidative damage, inflammation, telomere attrition and cell senescence 24. Interestingly, the inhibition of oxidative phosphorylation prevents the development of osimertinib resistance in EGFR-mutation positive NSCLC 25. The anticancer potential of pterostilbene has been demonstrated in several tumor models, including lung cancer 14, 26 and TNBC 27. In the present work, although we were not able to reconfirm the inhibitory effect of pterostilbene alone on Src and MET activation 15, or other downstream components (**Figure 2C**), the combination of osimertinib plus pterostilbene was synergistic in five-, and downregulated the activation of STAT3, Src-YAP1 and CDCP1 in two EGFR-mutation positive NSCLC cell lines (**Figure 4B**).

Pterostilbene was shown to modulate tumor-associated macrophages (TAMs), by modulating MUC1.^28^ High MUC1 mRNA expression is correlated with poorer overall survival, and pterostilbene-based inhibition subverted the tumor-microenvironment to an anti-tumoral state by reducing stemness induction of TAMs. Therefore, MUC1 expression levels may predict which patients can benefit from pterostilbene treatment. Another important piece of information is that constitutively active STAT3 increases MUC1 expression levels on both the mRNA and protein level, explaining our synergistic in vitro results.^29^ Therefore, further investigation in terms of feasibility and in vivo experiments should be carried out.

We are currently performing a proof of concept single-center clinical study, based on the model depicted in **Figure 4B**. This study will assess if pterostilbene is an economical but highly hopeful approach to increase the antitumoral activity of EGFR inhibitors in EGFR-mutation positive NSCLC patients, and should clarify whether this combined treatment strategy can be safely incorporated into routine clinical practice.

## Figures and Tables

**Table 1:** Used antibodies, including dilutions and catalog numbers.

Western Blotting Primary Antibody	Dilution	Company and Catalog number
Rabbit anti-AXL	1:1000	Cell Signaling (#8661)
Rabbit anti-Phospho-AXL (Y779)	1:200	R&D Systems (#AF2228)
Mouse anti-Beta-actin	1:5000	Sigma Aldrich (#A5441)
Rabbit anti-CDCP1	1:1000	Cell Signaling (#4115)
Rabbit anti-Phospho-CDCP1 (Y707)	1:1000	Cell Signaling (#13111)
Rabbit anti-ERK1/2	1:1000	Cell Signaling (#9102)
Rabbit anti-Phospho-ERK1/2 (T202/Y204)	1:1000	Cell Signaling (#9101)
Rabbit anti-MET	1:1000	Cell Signaling (#8198)
Rabbit anti-Phospho-MET (Y1234/1235)	1:500	Cell Signaling (#3077)
Rabbit anti-Src	1:1000	Cell Signaling (#2109)
Rabbit anti-Phospho-Src Family (Y416)	1:1000	Cell Signaling (#6943)
Mouse anti-STAT3	1:1000	Cell Signaling (#9139)
Rabbit anti-Phospho-STAT3 (Y705)	1:1000	Cell Signaling (#9145)
Mouse anti-YAP1	1:1000	Cell Signaling (#12395)
Rabbit anti-Phospho-YAP1 (Y357)	1:1000	Abcam (#ab62751)
HRP-linked goat anti-rabbit (from donkey)	1:5000	GE Healthcare Life Sciences (NA934-1ML)
HRP-linked goat anti-mouse (from sheep)	1:5000	GE Healthcare Life Sciences (NA931-1ML)

## Abbreviations

ATCC: american type culture collection
CDCP1: cub domain-containing protein-1
CI: combination index
DMSO: dimethylsulfoxide
EGFR: epidermal growth factor receptor
ER: endoplasmic reticulum
FAK: focal adhesion kinase
FBS: fetal bovine serum
HRP: horseradish peroxidase
IC50: the half maximal inhibitory concentration
JAK2: janus kinase 2
MTT: 3-[4,5-dimethylthiazol-2-yl]-2,5-diphenyltetrazolium bromide
NSCLC: non-small cell lung cancer
PM: pterocarpus marsupium
PVDF: polyvinylidene difluoride
RIPA: radioimmunoprecipitation assay
RTKs: receptor tyrosine kinases
SDS: sodium dodecyl sulfate
SFKs: src family kinases
STAT3: signal transducer and activator of transcription 3
TAMs: tumor-associated macrophages
TKIs: tyrosine kinase inhibitors
TNBC: triple negative breast cancer
YAP1: src-yes-associated protein 1
95%CI: 95% confidence interval

## References

[1] Sordella R, Bell DW, Haber DA, Settleman J (2004). Gefitinib-sensitizing EGFR mutations in lung cancer activate anti-apoptotic pathways. Science.

[2] Rosell R, Carcereny E, Gervais R, Vergnenegre A, Massuti B, Felip E (2012). Erlotinib versus standard chemotherapy as first-line treatment for European patients with advanced EGFR mutation-positive non-small-cell lung cancer (EURTAC): a multicentre, open-label, randomised phase 3 trial. Lancet Oncol.

[3] Gao SP, Mark KG, Leslie K, Pao W, Motoi N, Gerald WL (2007). Mutations in the EGFR kinase domain mediate STAT3 activation via IL-6 production in human lung adenocarcinomas. J Clin Invest.

[4] Fan W, Tang Z, Yin L, Morrison B, Hafez-Khayyata S, Fu P (2011). MET-independent lung cancer cells evading EGFR kinase inhibitors are therapeutically susceptible to BH3 mimetic agents. Cancer Res.

[5] Lee HJ, Zhuang G, Cao Y, Du P, Kim HJ, Settleman J (2014). Drug resistance via feedback activation of Stat3 in oncogene-addicted cancer cells. Cancer Cell.

[6] Zhong Z, Wen Z, Darnell JE Jr (1994). Stat3: a STAT family member activated by tyrosine phosphorylation in response to epidermal growth factor and interleukin-6. Science.

[7] Nan J, Du Y, Chen X, Bai Q, Wang Y, Zhang X (2014). TPCA-1 is a direct dual inhibitor of STAT3 and NF-kappaB and regresses mutant EGFR-associated human non-small cell lung cancers. Mol Cancer Ther.

[8] Chaib I, Karachaliou N, Pilotto S, Codony Servat J, Cai X, Li X (2017). Co-activation of STAT3 and YES-Associated Protein 1 (YAP1) Pathway in EGFR-Mutant NSCLC.

[9] Codony-Servat C, Codony-Servat J, Karachaliou N, Molina MA, Chaib I, Ramirez JL (2017). Activation of signal transducer and activator of transcription 3 (STAT3) signaling in EGFR mutant non-small-cell lung cancer (NSCLC). Oncotarget.

[10] Karachaliou N, Chaib I, Cardona AF, Berenguer J, Bracht JWP, Yang J (2018). Common Co-activation of AXL and CDCP1 in EGFR-mutation-positive Non-smallcell Lung Cancer Associated With Poor Prognosis. EBioMedicine.

[11] Rosell R, Karachaliou N, Cui JJ, Chaib I, Berenguer J, Bratch J (2017). P3.01-073 TPX-0005 with an EGFR Tyrosine Kinase Inhibitor (TKI) Overcomes Innate Resistance in EGFR Mutant NSCLC. Journal of Thoracic Oncology.

[12] McCormack D, McFadden D (2013). A review of pterostilbene antioxidant activity and disease modification. Oxid Med Cell Longev.

[13] Tolba MF, Abdel-Rahman SZ (2015). Pterostilbine, an active component of blueberries, sensitizes colon cancer cells to 5-fluorouracil cytotoxicity. Sci Rep.

[14] Ma Z, Yang Y, Di S, Feng X, Liu D, Jiang S (2017). Pterostilbene exerts anticancer activity on non-small-cell lung cancer via activating endoplasmic reticulum stress. Sci Rep.

[15] Su CM, Lee WH, Wu AT, Lin YK, Wang LS, Wu CH (2015). Pterostilbene inhibits triple-negative breast cancer metastasis via inducing microRNA-205 expression and negatively modulates epithelial-to-mesenchymal transition. J Nutr Biochem.

[16] Ruiz MJ, Fernandez M, Pico Y, Manes J, Asensi M, Carda C (2009). Dietary administration of high doses of pterostilbene and quercetin to mice is not toxic. J Agric Food Chem.

[17] Kapetanovic IM, Muzzio M, Huang Z, Thompson TN, McCormick DL (2011). Pharmacokinetics, oral bioavailability, and metabolic profile of resveratrol and its dimethylether analog, pterostilbene, in rats. Cancer Chemother Pharmacol.

[18] Riche DM, Riche KD, Blackshear CT, McEwen CL, Sherman JJ, Wofford MR (2014). Pterostilbene on metabolic parameters: a randomized, double-blind, and placebo-controlled trial. Evid Based Complement Alternat Med.

[19] Chou TC (2010). Drug combination studies and their synergy quantification using the Chou-Talalay method. Cancer Res.

[20] Narayan RS, Fedrigo CA, Brands E, Dik R, Stalpers LJ, Baumert BG (2017). The allosteric AKT inhibitor MK2206 shows a synergistic interaction with chemotherapy and radiotherapy in glioblastoma spheroid cultures. BMC Cancer.

[21] Yoshida T, Zhang G, Smith MA, Lopez AS, Bai Y, Li J (2014). Tyrosine phosphoproteomics identifies both codrivers and cotargeting strategies for T790M-related EGFR-TKI resistance in non-small cell lung cancer. Clin Cancer Res.

[22] Ichihara E, Westover D, Meador CB, Yan Y, Bauer JA, Lu P (2017). SFK/FAK Signaling Attenuates Osimertinib Efficacy in Both Drug-Sensitive and Drug-Resistant Models of EGFR-Mutant Lung Cancer. Cancer Res.

[23] Murakami Y, Sonoda K, Abe H, Watari K, Kusakabe D, Azuma K (2017). The activation of SRC family kinases and focal adhesion kinase with the loss of the amplified, mutated EGFR gene contributes to the resistance to afatinib, erlotinib and osimertinib in human lung cancer cells. Oncotarget.

[24] Li YR, Li S, Lin CC (2018). Effect of resveratrol and pterostilbene on aging and longevity. Biofactors.

[25] Martin MJ, Eberlein C, Taylor M, Ashton S, Robinson D, Cross D (2016). Inhibition of oxidative phosphorylation suppresses the development of osimertinib resistance in a preclinical model of EGFR-driven lung adenocarcinoma. Oncotarget.

[26] Wang YJ, Lin JF, Cheng LH, Chang WT, Kao YH, Chang MM (2017). Pterostilbene prevents AKT-ERK axis-mediated polymerization of surface fibronectin on suspended lung cancer cells independently of apoptosis and suppresses metastasis. J Hematol Oncol.

[27] Wakimoto R, Ono M, Takeshima M, Higuchi T, Nakano S (2017). Differential Anticancer Activity of Pterostilbene Against Three Subtypes of Human Breast Cancer Cells. Anticancer Res.

